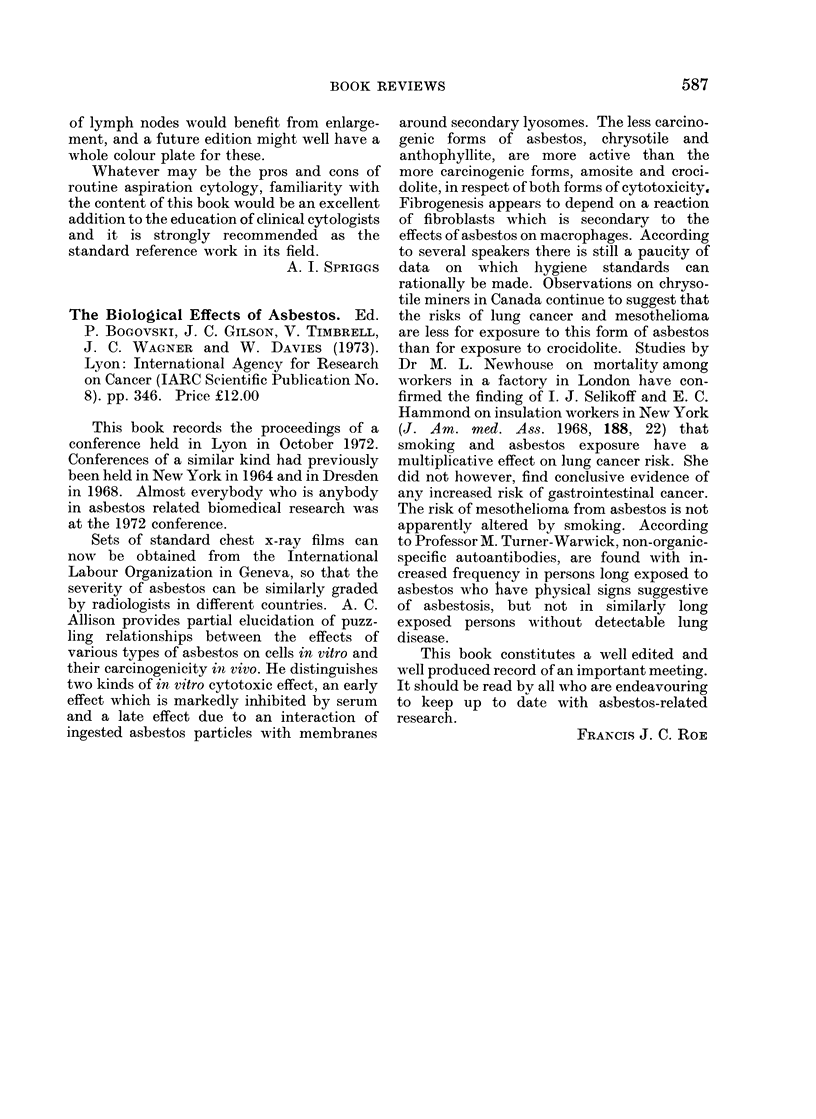# The Biological Effects of Asbestos

**Published:** 1974-12

**Authors:** Francis J. C. Roe


**The Biological Effects of Asbestos.** Ed. P. BOGOVSKI, J. C. GILSON, V. TIMBRELL, J. C. WAGNER and W. DAVIES (1973). Lyon: International Agency for Research on Cancer (IARC Scientific Publication No. 8). pp. 346. Price £12.00

This book records the proceedings of a conference held in Lyon in October 1972. Conferences of a similar kind had previously been held in New York in 1964 and in Dresden in 1968. Almost everybody who is anybody in asbestos related biomedical research was at the 1972 conference.

Sets of standard chest x-ray films can now be obtained from the International Labour Organization in Geneva, so that the severity of asbestos can be similarly graded by radiologists in different countries. A. C. Allison provides partial elucidation of puzzling relationships between the effects of various types of asbestos on cells *in vitro* and their carcinogenicity *in vivo*. He distinguishes two kinds of *in vitro* cytotoxic effect, an early effect which is markedly inhibited by serum and a late effect due to an interaction of ingested asbestos particles with membranes around secondary lyosomes. The less carcinogenic forms of asbestos, chrysotile and anthophyllite, are more active than the more carcinogenic forms, amosite and crocidolite, in respect of both forms of cytotoxicity. Fibrogenesis appears to depend on a reaction of fibroblasts which is secondary to the effects of asbestos on macrophages. According to several speakers there is still a paucity of data on which hygiene standards can rationally be made. Observations on chrysotile miners in Canada continue to suggest that the risks of lung cancer and mesothelioma are less for exposure to this form of asbestos than for exposure to crocidolite. Studies by Dr M. L. Newhouse on mortality among workers in a factory in London have confirmed the finding of I. J. Selikoff and E. C. Hammond on insulation workers in New York (*J. Am. med. Ass.* 1968, **188**, 22) that smoking and asbestos exposure have a multiplicative effect on lung cancer risk. She did not however, find conclusive evidence of any increased risk of gastrointestinal cancer. The risk of mesothelioma from asbestos is not apparently altered by smoking. According to Professor M. Turner-Warwick, non-organic-specific autoantibodies, are found with increased frequency in persons long exposed to asbestos who have physical signs suggestive of asbestosis, but not in similarly long exposed persons without detectable lung disease.

This book constitutes a well edited and well produced record of an important meeting. It should be read by all who are endeavouring to keep up to date with asbestos-related research.

FRANCIS J. C. ROE